# A model of cue integration as vector summation in the insect brain

**DOI:** 10.1098/rspb.2023.0767

**Published:** 2023-06-28

**Authors:** Robert Mitchell, Shahrzad Shaverdian, Marie Dacke, Barbara Webb

**Affiliations:** ^1^ Institute for Perception, Action, and Behaviour, The University of Edinburgh School of Informatics, Edinburgh, Edinburgh EH8 9AB, UK; ^2^ Lund Vision Group, Department of Biology, Lund University, Lund SE-223 62, Sweden

**Keywords:** vector, cue integration, plasticity, contrast, reliability, neural model

## Abstract

Ball-rolling dung beetles are known to integrate multiple cues in order to facilitate their straight-line orientation behaviour. Recent work has suggested that orientation cues are integrated according to a vector sum, that is, compass cues are represented by vectors and summed to give a combined orientation estimate. Further, cue weight (vector magnitude) appears to be set according to cue reliability. This is consistent with the popular Bayesian view of cue integration: cues are integrated to reduce or minimize an agent's uncertainty about the external world. Integration of orientation cues is believed to occur at the input to the insect central complex. Here, we demonstrate that a model of the head direction circuit of the central complex, including plasticity in input synapses, can act as a substrate for cue integration as vector summation. Further, we show that cue influence is not necessarily driven by cue reliability. Finally, we present a dung beetle behavioural experiment which, in combination with simulation, strongly suggests that these beetles do not weight cues according to reliability. We suggest an alternative strategy whereby cues are weighted according to relative contrast, which can also explain previous results.

## 1. Introduction

Cue integration is the process of combining multiple redundant sources of information to form a single estimate of a property of the world [1]. A widespread assumption in the cue integration literature is that brains integrate cues for the *purpose* of maximizing certainty about the estimate [2], from which it follows that cues should be weighted according to their reliability [1,3]. Integration of angular cues (e.g. for orientation) has previously been formalized as a vector sum [4]—if different directional cues are represented as vectors with magnitudes reflecting their weighting, the integration is given by their sum.

Recent models of insect navigation have suggested that vector computation plays a major role in their behavioural capabilities [5,6] and more specifically that they perform reliability-weighted vector-based cue integration [7–9]. This idea has been bolstered by direct evidence that the insect central complex has the necessary circuit properties to support vector computations by representing vectors as sinusoid curves (vector phasor representation) in activity across a neural array [10]. The central complex (CX) is a collection of midline neuropils which is highly conserved across insect species [11], of which the key components are the protocerebral bridge (PB), ellipsoid body (EB), fan-shaped body and a pair of noduli [12,13]. The function and structure of the CX has so far been mapped in most detail in *Drosophila*. The head direction of the insect with respect to external cues is tracked by the activity of a set of EB neurons known as E-PGs [13,14], also colloquially referred to as ‘compass neurons’. E-PGs receive input from structures known as the bulbs which sit laterally to the CX [13,15–17] and the bulbs house a class of neurons known as ring neurons (named for their ring-like arborizations in the EB [12]). A subset of ring neurons, the R neurons, are directionally tuned, responding to the position of a cue around the animal [18]. Each R neuron connects to all E-PGs [13,19] and these connections are plastic, enabling flexible remapping of the outside world onto the compass neurons [16,20,21]. Ring neurons also cluster into different types which seem to encode the position of different orientation cues [15,17]. Where known, the functional role of different CX structures appears to be preserved in other insects. Given that R neurons are directionally tuned, we propose that their population activity profile could be roughly sinusoidal in shape, with the consequence that the E-PG's activity could be a pointwise sum of sinusoids, representing a vector sum of different cue modalities. The encoding of different cue modalities as well as the ability to create flexible relationships between available cues make the R-to-E-PG interface an ideal substrate for flexible multimodal cue integration [9,13,19].

Multimodal cue integration has recently been discovered in the ball-rolling dung beetle *Kheper lamarcki* (MacLeay, 1821) [22]. These beetles have long been known to have a variety of orientation cues at their disposal [23] which enable them to maintain a straight path of arbitrary direction when rolling a ball away from the dung pile. However, it was previously thought that these beetles followed a cue hierarchy [24], i.e. a single cue would dominate at any time, depending on circumstances such as availability, evolutionary history, etc. A collection of new studies are showing that these beetles can in fact use multiple cues simultaneously [9,22,25,26]. The most recent study which examined the cue integration of *K. lamarcki* suggested that these beetles followed a vector summation strategy for the integration of (the directional information provided by) a visual sun cue and a mechanosensory wind cue [9]. Both visual and mechanosensory cues are known to reach the ring neurons in flies [17], with visual input also being confirmed in dung beetles [27]. *Kheper lamarcki* has also been shown to be able to synchronize information from different compass cues [22]. Plasticity between the ring neurons and E-PGs could enable such synchronization [16,17,19,20]. Angular velocity appears to be the signal that regulates plasticity between the ring neurons and E-PG neurons [21] (also modelled by [28]). It is therefore striking to note that *K. lamarcki* perform a stereotyped ‘orientation dance’ before beginning ball-rolling behaviour [29] which takes the form of a rotation on top of the ball, and ignore orientation cues not present during the dance [30]. Thus, dung beetle behaviour aligns well with known and plausible functionality of the insect head direction circuit in the CX.

In this paper, we extend the model of the insect head direction circuit from [31,32] by including the R neurons and their plastic connections onto E-PG neurons [15–17,20]. We show that if plasticity is gated by angular velocity, the connections form coherently and allow the circuit to perform cue integration by vector summation. We then explore whether and how the relative reliability of cues determines their influence on behaviour, in simulations of and experiments on dung beetles. Together, these results strongly suggest that dung beetles do not weight cues according to actual reliability. We propose that they instead use cue contrast as a proxy estimate for reliability.

## 2. Material and methods

### (a) Conceptual framework

**Figure 1.**
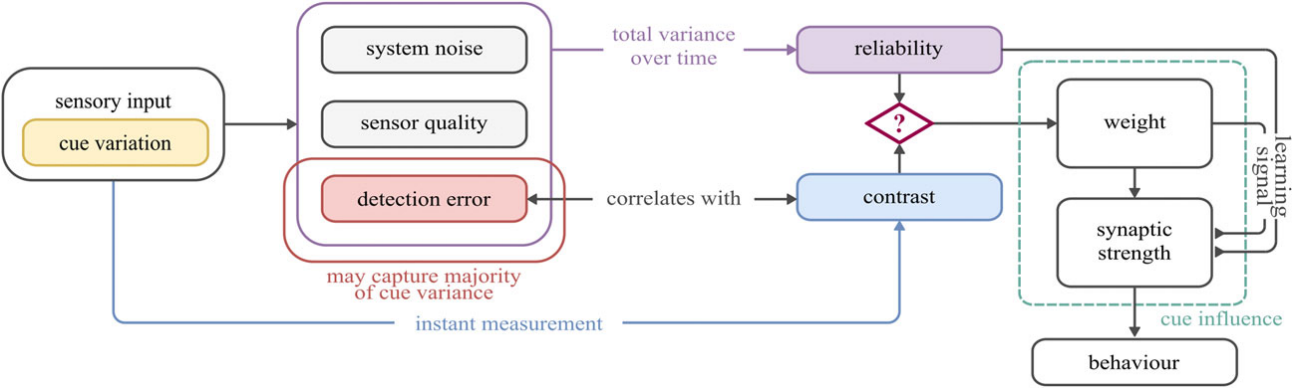
Concepts in cue integration. *Reliability* describes the variance of a cue estimate over time, reflecting external cue variation, but also noise or limitations in the sensory processing system. *Contrast* of a cue with the background influences its detection (and hence reliability) but is an instantaneous property. *Weight*, in this paper, strictly refers to the amplitude of the sinusoid that represents each directional cue at input to the integration circuit. *Synaptic strength* describes the strength of the connections between neurons. Synaptic strength is modified during learning and (as we will show) can be affected differently by cue weight and cue reliability. Cue influence describes the combined effect of weight and the synaptic strengths on final behavioural output.

In figure 1 we provide clarification of how several otherwise ambiguous terms will be used in this paper.

### (b) Circular cue integration as a vector sum

Consider two von Mises random variables *C* and *H*. For samples *c_i_* and *h_i_*, their integration is given by [4]:

2.1 $$l_{i}=h_{i}+\mathrm{atan}2\left(\sin(c_{i}-h_{i}),\left(\frac{\kappa_{H}}{\kappa_{C}}\right)+\cos(c_{i}-h_{i})\right)\;,$$

with the joint reliability given by

2.2 $$\kappa_{L}=\sqrt{\kappa_{H}^{2}+\kappa_{C}^{2}+2\kappa_{H}\kappa_{C}\text{cos}(c_{i}-h_{i})},$$

*κ_H_*, *κ_C_* could be arbitrary positive weights, but for ‘optimal’ integration are set as the concentrations (reliabilities) of the von Mises distributions which describe *C* and *H*. Note, however, this ‘optimality’ is based on approximation to the classic maximum-likelihood model [1] for specific weight combinations and conflicts (see appendix of [4]).

This model for integration is equivalent to polar vector addition. Given two cue vectors $\bar{c}=(c_{i},\kappa_{C})$ and $\bar{h}=(h_{i},\kappa_{H})$, the integration is simply $\bar{l}=\bar{c}+\bar{h}=(l_{i},\kappa_{L})$, where *l_i_* gives the angle and *κ*_L_ the joint reliability. Our aim in the following is to construct a neural circuit that results in equivalent integration. More specifically, it should produce an output corresponding to the angle, *l_i_*, which can be used to guide steering behaviour; we do not require the magnitude *κ*_L_ in our neural model to match that of the pure vector model.

### (c) Computer modelling

#### (i) Ring model overview

Here we provide a textual overview of our model; the included neurons and how they interact. For implementation details, please refer to our electronic supplementary material or codebase. We use fruit fly nomenclature throughout, but non-fly homologues [33] are given for each neuron class.

Our model is a hybrid of the biologically constrained models of the head direction circuits of the fruit fly *Drosophila melanogaster* and the desert locust *Schistocerca gregaria* presented in a previous comparative study [31]; similar to another presented in the context of a model of ant orientation [32]. Specifically, this means we use the ‘locust’ 8-fold columnar structure (consistent with the description of the PB of *K. lamarcki* [34]) but the ‘fly’ uniform inhibition [31,35] between compass cells. While it is known that intra compass cell inhibition in beetles more closely reflects the locust anatomy [36], we use a hybrid structure to motivate general plausibility across insect species. The differences in connectivity do not create any functional differences in the circuit which are relevant to the cue integration problem [31]. We extend previous models [31,32] by including the R neurons and their plastic all-to-all connections onto the E-PGs. Our R neuron representation is based on descriptions given by [15–17,20]. Our neurons use the same basic firing rate encoding as [5].

**Figure 2.**
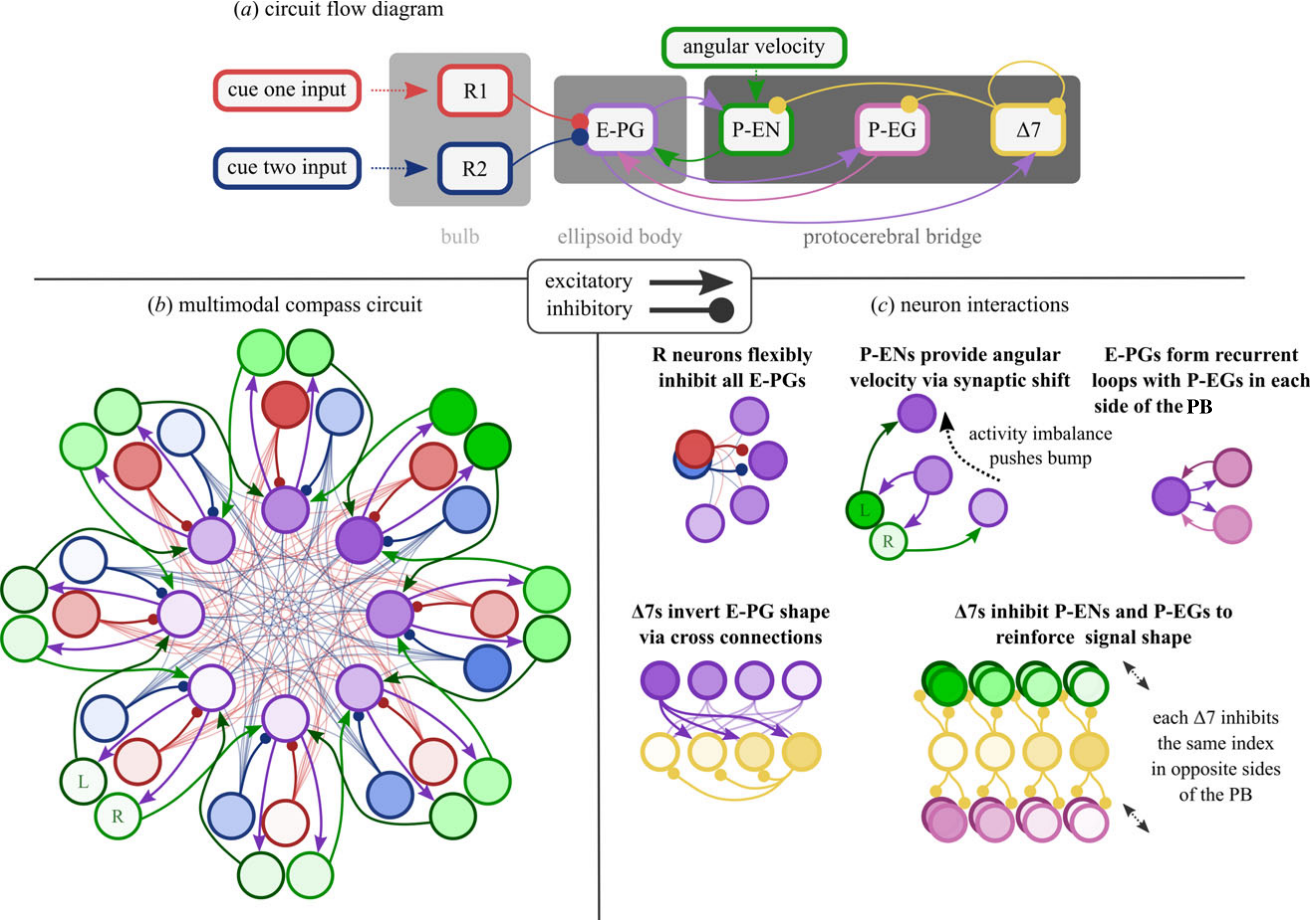
Model illustration. (*a*) High-level view of information flow through the network (also see the electronic supplementary material, figure S1). (*b*) The multimodal compass circuit which is the focus of this paper. Each R neuron population (blue, red) encodes a different cue modality as a sinusoidal bump (indicated by shading). R neurons provide input to E-PGs (purple) via plastic all-to-all connections which are formed in combination with angular velocity from P-EN neurons (green). (*c*) Illustrative microcircuits describing how the different neuron populations interact throughout the model. PB, protocerebral bridge.

*R (TL) neurons.* R neurons function as the cue inputs and are split into two groups, one for each cue: R_1_ and R_2_ (note that these are just indices, we are not explicitly modelling specific classes of ring neuron). Each R neuron has a receptive field centred on its preferred direction. The distribution of receptive fields results in a sinusoidal activity pattern across each R neuron group; the phase of the sinusoid encodes the angle to the cue and the amplitude gives its weight. In the following work we use eight neurons per group to represent the cardinal and ordinal directions (this also makes visualizing the R → E-PG mappings easier—see below). As the exact number of R neurons per modality is still uncertain [13,37] we also provide a supplementary exploration of R neuron population size and how this affects the proposed function. In brief, we found that there is no significant difference in network function where R neuron groups are of equal size; however, where group sizes differed, the larger group received more influence in the resulting integration (see the electronic supplementary material).

*P-EN (CL2) neurons.* P-ENs function as self-motion inputs and are a source of local excitation for the E-PG neurons. The P-ENs are spread across the 16 columns of the PB, dividing into two sub-populations of eight, which represent the same directional signal (bump) twice over [31,38]; from our observations, this bump appears to emerge owing to input from the E-PGs and Δ7s. The different sub-populations drive the E-PG bump in different directions via a single-column synaptic shift. The difference in activity between the two sub-populations is thought to encode idiothetic angular velocity, though it is not currently known if this is proprioception-based or motor efference [21,38]. Total P-EN activity is described as relatively constant across all 16 neurons [38]. We found that the relative constancy of the overall activity was critical in ensuring that the E-PGs are not saturated or starved when the network experiences different angular velocities.

*E-PG (CL1a) neurons.* E-PGs receive cue azimuth information from the R neurons, self-motion information and local excitation from the P-ENs, and recurrent excitation from the P-EGs. The E-PGs are typically described as dividing into 16 wedges which form a single bump of activity [14]. For ease of development and presentation we instead use eight neurons, however, the number should not significantly affect the accuracy of the angle stored.

In order to account for the results of [14] (E-PG bump retention in darkness and E-PG visual capture) as well as our own proposed learning routine (where E-PGs are driven primarily by angular velocity input), input to the E-PGs is context dependent. If learning is taking place, then R neuron input to the E-PGs is reduced. This can be thought of as an inhibition of R neurons (or enhancement of P-EN neurons) which occurs when the agent/animal is attempting to learn about the cues (see Discussion).

Δ7*(TB1) neurons.* Δ7s only receive input from the E-PGs. They: (i) invert the bump from the E-PGs and feed this back into the PB, and (ii) inhibit other Δ7s. Both features appear to aid the stability of the bump in the E-PGs and as a result, the rest of the circuit.

*P-EG (CL1b) neurons.* Finally, the P-EGs provide a simple recurrent loop with the E-PGs, moderated by the Δ7s. Together with the P-ENs, the P-EGs maintain activity in the E-PGs in the absence of external input.

#### (ii) R-to-E-PG connections

Our model is initialised using a default R-to-E-PG connection pattern (figure 4*b*; electronic supplementary material); once the model is initialised, R-to-E-PG connections are all set to an equal value and learned from scratch. In our model, plasticity between R and E-PG neurons is only enabled during specific learning events. R →E-PG connections are learned using the following anti-Hebbian update rule:

2.3 $$\Delta w_{i,j}=-\eta \cdot (r_{R_{l,j}}-\theta_{R_{l}})\cdot (r_{E-PG_{i}}-\theta_{E}),$$

where *w_i_*_,*j*_ is the synaptic strength from from *R_l_*_,*j*_ onto E-PG*_i_*, *θ_E_* = 0.9 is the threshold for E-PG activity, and *η* = 0.1 is the learning rate. $\theta_{R_{l}}$ is an adaptive threshold on R neuron activity with $\theta_{R_{l}}=0.7\cdot \max({\bar{r}}_{R_{l}})$ (a fraction of the maximum R neuron activation for group *l*). Note that if the sinusoidal shape of the R neuron population code can be assumed, then this adaptive threshold could equivalently be a fraction of total activity. Connections are then normalized such that the total synaptic strength *onto each* E-PG sums to 1. Hebbian learning requires a rapid compensatory mechanism in order to function correctly, and the post-synaptic normalization we are using (keeping total input constant) is one of a few potential regulatory mechanisms (see [39]).

#### (iii) Behavioural simulations

To provide additional insight into behavioural results, we also constructed a basic behavioural simulation. Conceptually, an agent is placed in the centre of a circular arena and tasked with walking to the edge (in an arbitrary direction with respect to external cues). At the start of each walk (or ‘roll’) the agent performs a learning rotation (or dance). On the first roll of an experiment, the dance will establish R → E-PG mappings from a blank mapping (all Rs connect to all E-PGs equally). Subsequent dances will update the existing map. Note that, while cue noise is included, self-motion noise is excluded during the dance. We assume that, over a short rotation, the agent's perceived motion is correct. Simulation configuration information is given alongside the relevant results. For full details of our simulation environment, please see our electronic supplementary material or codebase.

### (d) Animal behaviour

#### (i) Beetle collection and husbandry

Using dung-baited pitfall traps, the diurnal, ball-rolling beetle *K. lamarcki* was collected at the game farm ‘Stonehenge’ in Vryburg, South Africa (24.32° E, 26.39° S) in November 2021, February 2022, and November 2022. Behavioural experiments were conducted in Lund, Sweden from December 2021 to May 2022, and during December 2022. All beetles were stored in opaque, plastic bins filled with sand of a consistency similar to their natural soil, and fed with horse dung 2–4 times per week. Prior to each experiment, beetles were removed from the sand filled bins and placed in a separate box containing fresh dung for them to construct into balls. Beetles that began to roll their balls of dung were used for experimentation.

#### (ii) Behavioural experiments

**Figure 3.**
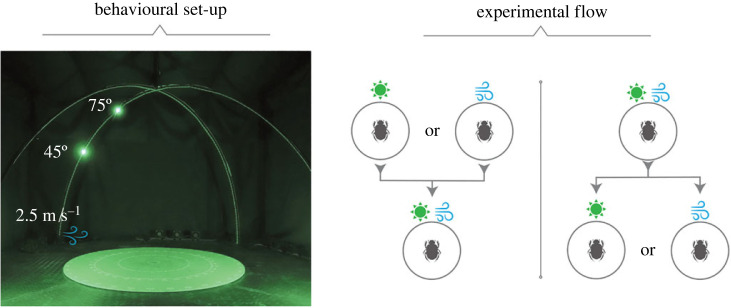
Experimental set-up and experimental flow.

*Experimental set-up.* All experiments were conducted in an indoor set-up consisting of two metal arches which were crossed to form the skeleton of a hemisphere (*r* = 1.5 m). Each arch was lined with 141 LEDs (520 nm, DotStar; Adafruit Industries, New York, USA) where individual LEDs served as ersatz sun cues with an intensity of 2 × 10^11^ photons cm^−2^ s^−1^ (QE65000; Ocean Optics) as measured from the centre of the set-up at a height of about 7 cm (corresponding to the approximate height of a beetle when on top of its dung ball). Under the arches, in the centre of the set-up, there was a circular, sand-painted arena (radius = 0.3 m); the perimeter of the arena was marked in five degree increments (from 0–355°) and 0° was aligned with magnetic north. Finally, a wind generator was positioned 1.3 m from the centre of the arena and aligned with one arm of the hemisphere. The generator consisted of three fans (PFR0912XHEE, 4.50 *A*; Delta Electronics Inc., Taipei City, Taiwan) distributed evenly over 1.0 m, and was configured to create an air current with a speed of 2.5 m s^−1^ when measured from the centre of the arena. The elevation of the ersatz sun and the wind speed were controlled using custom-built software in conjunction with a Raspberry Pi 4 Model B. All experiments were filmed using a Sony camera (FDRAX53 Handycam) mounted on a tripod above the arena. The set-up was constructed inside a 3 × 3 m tent constructed from blackout cloth (figure 3*a*).

*Experimental procedure.* A beetle was placed with its ball in the centre of the circular arena and allowed to roll the ball to the perimeter where its exit angle was noted. The beetle was placed back in the centre and the procedure was repeated to obtain 10 exits in the presence of either an isolated sun or wind cue, or both cues in alignment. When a beetle started with a single cue in isolation, the unseen cue would be added for the subsequent 10 rolls (i.e. if the beetle starts with a sun cue, we add a wind cue). When a beetle started with both cues, we removed one of the cues (figure 3*b* for schematic overview). In total, each beetle rolled its ball 20 times. This experimental procedure was carried out at solar elevations of 45° and 75°, with a wind speed of 2.5 m s^−1^ (based on [9]). The initial condition was presented in a pseudo-randomized order.

*Statistical analysis.* To assess the orientation precision of an individual beetle, the mean vector length (*r*-value) was calculated from 10 exit bearings. To test for significant differences between populations of *r*-values, paired Wilcoxon (Wilcoxon signed-rank) tests were used. Statistical analyses were performed using Oriana 3.21 (Kovach Computing Services, Anglesey, UK) and RStudio 4.1.0. [40]. All *p*-values presented are unadjusted.

## 3. Results

### (a) The network approximates the angular component of a vector sum

**Figure 4.**
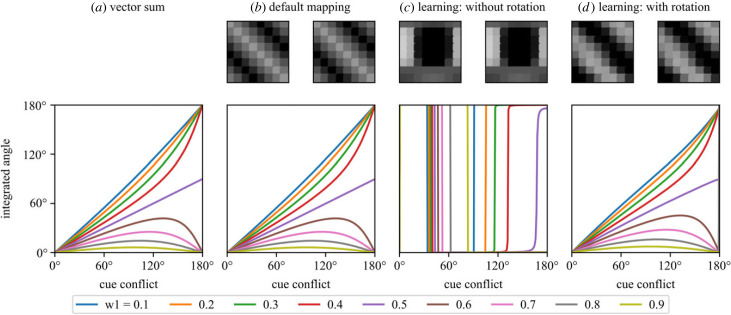
Integrated angular outputs for varying degrees of cue conflict. The pure vector sum [4] is compared against three instances of the ring model with different R → E-PG mappings (adjacency matrices shown in the top row). (*a*) Vector sum (equation (2.1)). (*b*) Default mapping which implements a pointwise sum-of-sinusoids. (*c*) The result of Hebbian plasticity alone (equation (2.3)). (*d*) The result of Hebbian plasticity combined with rotation; the agent rotates through 360° during learning.

A previous study [9] suggested that the insect head direction circuit could act as a substrate for vector summation, making it a likely location for compass cue integration. It is clear that our model circuit can encode the angular component of a vector sum to produce an integrated estimate from two directional cues as the amount of conflict between the directions they indicate, and their relative weight, is varied (compare figure 4*a*, *b*, and *d*). Setting a default diagonal connection pattern closely approximates the pure vector sum and our learning rule (equation (2.3)), in combination with a dance rotation, produces a similar diagonal pattern (with arbitrary offset) which generates qualitatively similar output. Learning without experiencing any rotation does not generate a diagonal connection pattern and clearly does not approximate the vector sum (figure 4*c*); rotation is required to form useful connections (see [21]).

### (b) Weight and reliability may independently affect cue influence

#### (i) Information about cue-state is stored in the R → E-PG mappings

It is clear from figure 4 that cue state during the learning routine has an effect on the mappings which are learned, which will ultimately affect behaviour. To investigate this further, we examined four different learning scenarios: cues separated, one cue useful, effect of weight, and effect of reliability (recall our definitions, figure 1).

**Figure 5.**
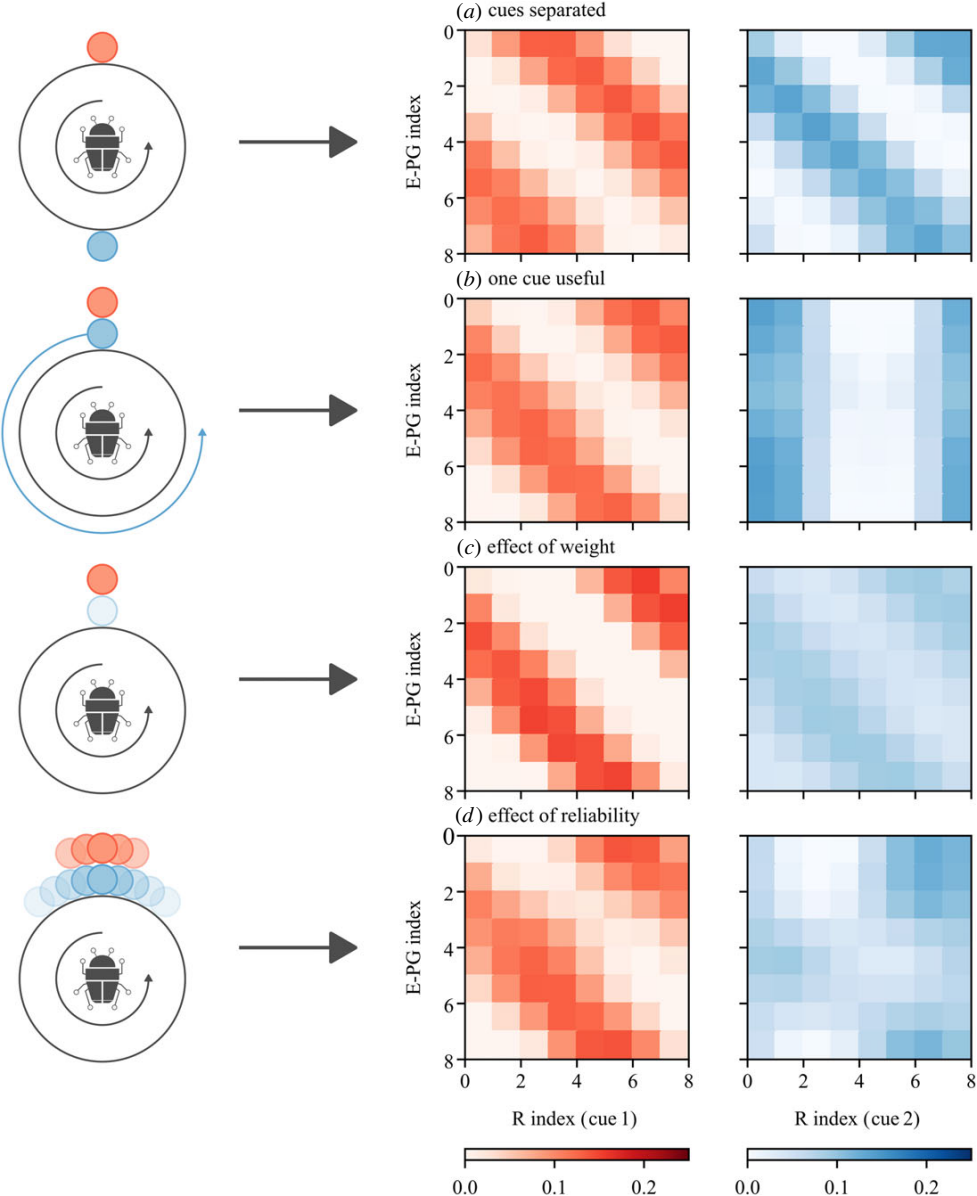
A selection of mappings learned for different cue configurations. (*a*) Each cue is equally weighted but they are physically separated by 179°. (*b*) Each cue is equally weighted, however the blue cue rotates with the agent (does not provide directional information). (*c*) In this case both cues are aligned, however the blue cue is one-quarter the weight of the red. (*d*) Cues are aligned and of equal weight but have different additive noise distributions (both von Mises). The red cue has concentration *κ*_red_ = 2 and the blue has *κ*_blue_ = 0.5; i.e. the cues have different reliabilities.

*Cues separated.* One very good reason to have all-to-all plastic connections between the Rs and E-PGs is that it potentially allows an agent to learn about the spatial relationship of physically separated cues [17,30]. Orientation cues may be azimuthally distant without being ‘in conflict’. We found that our model was able to encode cue offsets in the R → E-PG maps (figure 5*a*). The mapping for the second cue is offset from that of the first, meaning that the peak in each R group will be mapped to the same E-PG neuron. This ‘mental’ alignment indicates that the circuit could account for the modality transfer results seen by [22] (see behavioural simulations in the electronic supplementary material).

*One cue useful.* Here, we wanted to see what would happen in the case that one cue was given significant weight (*w*_blue_ = 0.5), but provided no directional information. A usable (diagonal) mapping is learned for the useful cue (red—figure 5*b*) but not for the other (blue). Note that nevertheless the synaptic strengths have a similar range. This indicates that a cue could provide significant input to the integration, even if it does not provide useful orientation information at the time it is learned.

*Effect of weight.* Here we see that cue weight at the time of learning is encoded in the mapping; greater weight leads to greater synaptic strength. In the extreme, a cue which was not present during the dance should have no influence on behaviour, as seen in the dung beetles [30].

*Effect of reliability.* In all previous experiments, cues have been noiseless (perfectly reliable). This is useful for exploration but may not reflect reality. Variation in the relationship between perceived cue motion and angular velocity should degrade the diagonal pattern of the mapping; this is evident from figure 4*c* and figure 5*b*. To explore the effect of this variation, we added von Mises noise to both cues with zero mean and concentrations *k*_red_ = 2 and *k*_blue_ = 0.5. These concentrations were chosen so that the effect of noise would be clearly visible. For higher concentration, the effect of noise on the mapping is minimal. For lower concentrations, it is clear that noise corrupts the mapping but does not appear to have a significant effect on the strength of the connections. Despite being noisy, it appears that the blue cue may have significant influence.

#### (ii) Cue influence is primarily governed by weight

**Figure 6.**
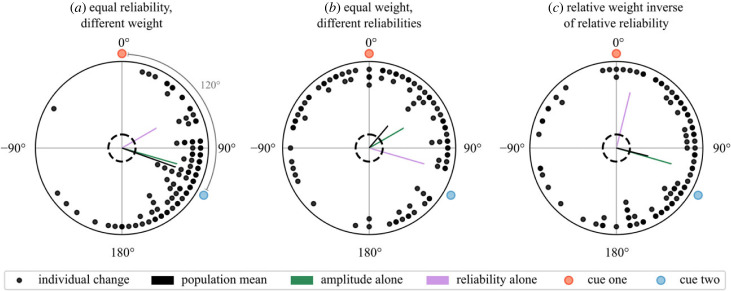
Effect of weight and reliability on cue influence in behavioural simulation of cue conflict. The population mean vector (black) falls closer to the theoretical vector sum where magnitudes are determined by cue weight (green) than where magnitudes are determined by reliability (magenta). Dashed rings indicate the threshold for significance using a Rayleigh test (*p* < 0.05).

The mapping results above indicated that both weight and reliability may affect synaptic strength and that the resulting influence on behaviour may not be easy to predict. We therefore simulated a series of cue conflict experiments to investigate the effects of weight and reliability on behavioural output. In each scenario (figure 6*a,c*), each agent (*n* = 100) exits the arena four times. Note that the agent will update its R → E-PG mappings each time it is placed in the arena centre (§2c(iii)). During the first three rolls the cues are aligned, and on the fourth they are set in conflict. Figure 6 shows the change in bearing between rolls three and four for each agent (as a black dot). Each change is indicative of cue influence for that agent and the population mean gives the average effect (as in the conflict experiment from [9]). The simulated population mean is compared to means predicted by pure vector summation where vector magnitudes are set based on cue reliability (figure 6, magenta vectors), or the arbitrary weight (sinusoidal amplitude) given to the cue (green vectors).

It is clear that the simulated behaviour more closely aligns with the results given by the vector sum where magnitudes are set according to cue weight; that is, sinusoidal amplitude primarily governs cue influence in the circuit. Reliability can affect influence on an individual level and we generally found these simulations to be quite variable (hence the large number of agents, also see the electronic supplementary material); however, this effect does not appear consistent enough to have a population-level effect. This strongly suggests that, if the R → E-PG interface does provide a substrate for cue integration as we propose, the amplitude of the R neuron response should change in response to some property of the cue in order to change cue influence (figure 1)

Previous studies in dung beetles have suggested that cue influence is governed by reliability, however, our simulated results indicate that this is not an emergent property of the head direction circuit combined with plasticity in R neuron connections. Rather, cue weight needs to be set explicitly, meaning it could be decoupled from reliability altogether.

### (c) Cue reliability does not determine cue weight in dung beetles

If dung beetles do weight orientation cues according to reliability then, according to the modelling provided by [9], beetle orientation precision with two cues should always be at least as good as orientation precision with the most reliable cue in isolation. We tested this prediction behaviourally using the same species, and used the neural model to perform a simulated version of the experiment to provide further insight.

#### (i) Animal behaviour

Beetles were tested in four scenarios:

(i) sun → sun and wind;
(ii) wind → sun and wind;
(iii) sun and wind → sun; and
(iv) sun and wind → wind.

**Figure 7.**
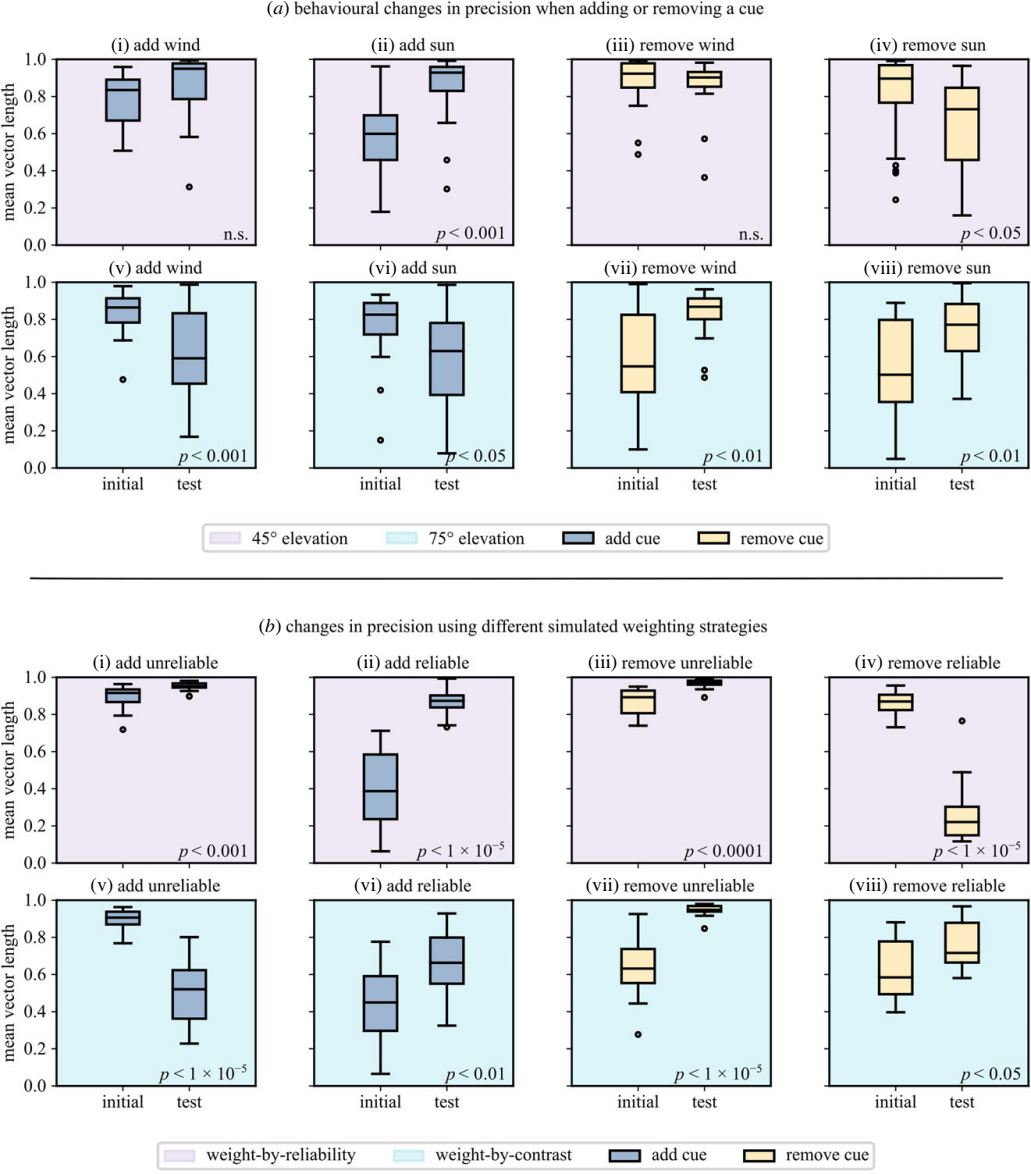
Changes in precision for single or multi-cue conditions. Bars give the median for each group, boxes give first and third interquartile range, and whiskers give the maximum and minimum values. Outliers are marked as separate data points. (*a*) Dung beetle orientation precision at 45° (top row, magenta) and 75° solar elevation (bottom row, cyan). (*b*) Simulated model precision using alternative weighting strategies. The top row (magenta) shows the expected outcome where cues are weighted by reliability, the bottom row (cyan) shows the expected outcome where cues are weighted by contrast (weight is decoupled from reliability). Reliable cue has concentration *κ* = 4, unreliable *κ* = 1.

In each scenario, we have an *initial* and a *test* condition. The beetles rolled their balls 10 times under the initial condition then 10 times under the test condition. The mean vector length is computed for each individual in each scenario for initial and test conditions and box plots are given in figure 7*a*. Each scenario was presented with a solar elevation of 45° or 75° (leading to eight scenarios overall). Note that when the initial condition consists of a single cue, this initial condition will replicate one of the single-cue conditions from [9] (specifically, figure 7*a*(i), (ii), (v) and (vi)). The same is not true when the single cue is presented in the test condition, as we assume that the order of presentation matters (see simulations below). Our key behavioural contribution is the quantitative comparison of single- and multi-cue precision, which was not discussed in [9] (and not explored in detail in [22]).

At a 45° solar elevation, when adding a wind cue to a sun cue, there was no significant difference in orientation precision between the two populations of *r*-values (*p* = 0.11, Wilcoxon signed-rank test; figure 7*a*(i), *n* = 20). By contrast, when a sun cue was added to a wind cue, there was a significant increase in the beetles' orientation precision (*p* < 0.001, Wilcoxon signed-rank test; figure 7*a*(ii), *n* = 20). When the beetles began with both a sun and wind cue and the wind was removed, there was no significant change in the orientation precision (*p* = 0.27, Wilcoxon signed-rank test; figure 7*a*(iii), *n* = 20). However, when the sun was removed, there was a significant decline in orientation precision (*p* < 0.05, Wilcoxon signed-rank test; figure 7*a*(iv), *n* = 30). In summary, at a 45° solar elevation, the orientation precision of the population of beetles *increased* when both sun and wind cues were present compared to a wind cue alone. Orientation precision with a sun cue alone was not significantly different from that with both cues present.

At a 75° solar elevation, when adding a wind cue to a sun cue, there was a significant decline in orientation precision (*p* < 0.01, Wilcoxon signed-rank test; figure 7*a*(v), *n* = 20). Similarly, orientation precision was reduced when a sun cue was added in combination with an existing wind cue (*p* < 0.05, Wilcoxon signed-rank test; figure 7*a*(vi), *n* = 20). By contrast, when removing the wind cue and leaving the sun cue in place—or vice versa—a significant increase in orientation precision was observed (*p* < 0.01, Wilcoxon signed-rank test; figure 7*a*(vii), *a*(viii), *n* = 20). In summary, at a 75° solar elevation, the orientation precision of the population of beetles *decreased* when both sun and wind cues were present. Two cues reduced orientation precision compared to one.

By comparing the sun-only conditions in figure 7 (*a*(i) and *a*(v), initial), we can see that the orientation precision does not change across the different elevations. This indicates that the reliability of the sun cue is not affected by this change in elevation. This is largely consistent with the findings of [9]; while they reported a slight reduction in precision from 45° to 75°, performance does not degrade significantly until elevations of 80° and above. Given that: (i) changing the elevation does not appear to change the reliability of the sun cue, (ii) changing the elevation from 45° to 75° changes the influence of a sun cue [9], and (iii) orientation precision decreases with multiple cues when the sun is at high elevations, it would appear that reliability is not the key component of cue influence for these beetles.

We suggest that the beetles use cue contrast in order to determine cue weight. Loosely, we reason that the contrast of a sun cue (intensity contrast between the solar and anti-solar hemispheres) drops as elevation increases which changes the relative weight compared to a wind cue with constant speed. As the relative contrast of the sun cue drops, the weight relationship changes but the reliabilities do not, meaning that the wind will receive a higher relative weight than the sun. As the wind is probably a less reliable cue (difficulty in reliable detection and turbulence), this weight change leads to a decrease in orientation performance. Note that our ersatz sun stimulus is monochromatic and its intensity is not varied over different elevations.

### (ii) Simulated behaviour

Using our neural circuit, we simulated two weighting strategies: weight-by-reliability and weight-by-contrast. Under weight-by-reliability, relative weight (relative sinusoidal amplitude) is given by the relative reliability of the cues:

3.1 $$w1=\frac{\kappa_{1}}{\kappa_{1}+\kappa_{2}}\;w2=\frac{\kappa_{2}}{\kappa_{1}+\kappa_{2}}$$

with *κ*_1_ = 4 and *κ*_2_ = 1 (w1 = 0.8, w2 = 0.2). Under weight-by-contrast the relative weight of the cue is set arbitrarily as the inverse of the relative reliability. We are not proposing a specific relationship between the two, only that they are not necessarily coupled.

In each case we simulate four scenarios, mimicking the behavioural assay:

(i) single reliable cue → add unreliable;
(ii) single unreliable cue → add reliable;
(iii) both cues → remove unreliable; and
(iv) both cues → remove reliable.

The agents roll 10 times under the initial condition and 10 times under the test condition; note that the R → E-PG mapping is not cleared between the initial and test conditions. This means that the order in which conditions are presented will affect the outcome. The results are shown in figure 7*b*; *p*-values indicate significance levels for a Wilcoxon signed-rank test between the initial and test conditions.

Following our reasoning above, at mid-elevations, the relative contrast and relative reliability of the two cues may be similar (the beetle data should appear to be weighted by reliability). At high elevations, the relative contrast of the light cue will drop but the relative reliability will remain unchanged, leading the unreliable wind cue to get more weight than it should (the beetle data should appear to be weighted by contrast).

Where cue weight is dictated by relative reliability (figure 7*b*, top row), our model generally matches the prediction that two cues should be better than one (median increase in orientation precision). While the change in simulated results is more extreme, we generally see the same pattern as in the beetles at 45° elevation (figure 7*a*, top row). Where we weight cues by (hypothetical) relative contrast, figure 7*b*(v), *b*(vii), and *b*(viii) indicate that two cues would be worse than one (decrease in median orientation precision). Again, the simulated mean vector length distributions are more extreme in their differences, however, the general pattern matches the beetle data at 75° elevation (figure 7*a*, bottom row). This reflects our broad expectation if cues are weighted according to relative contrast, as opposed to relative reliability. We propose that the beetles are weighting cues according to contrast, which aligns with reliability at 45° elevation, however, as elevation rises, contrast and reliability diverge leading to decreased multi-cue performance.

We note two anomalies in our simulated results. In figure 7*b*(iii), removing a cue makes the agents more precise where we would expect it to cause a decrease in performance. It is not clear why this is the case, but the effect is consistent across different random initialisations. Figure 7*b*(vi) presents another anomaly, the simulation reports an increase in precision where the analogous experiment from the beetle data (figure 7*a*(vi)) shows a decrease. Even when weighting cues arbitrarily, we would still expect adding a reliable cue to increase precision (in line with the simulation result). We are not sure why the beetles experience a decrease in orientation precision in this case. One possibility is that the biological data itself is highly variable. If we compare the ‘initial’ conditions in figure 7*a*(ii), *a*(vi) (where the beetles experienced wind in isolation), it is clear that the beetles are far more precise in the wind-only condition in figure 7*a*(vi), despite the fact that these conditions should be identical.

## 4. Discussion

The insect head direction circuit, specifically the EB, has been previously suggested to house a flexible multimodal compass [13,21]. Here we have provided a functional model which demonstrates that the EB can perform multimodal cue integration as vector summation. Importantly, the plasticity in mapping different cues (R neuron activity) to the EB during rotation establishes a common frame of reference for the vectors. The plasticity can also influence the relative contribution to the vector sum of the different cues. However, we show that this does not lead to an emergent reliability-based influence of the cues on the behaviour; some additional mechanism would be needed to set the input weights. This prompted an exploration into what determines cue weight in our model species, the dung beetle *K. lamarcki*. Contrary to previous studies [9,22,26], we found that these beetles do not appear to weight cues according to their reliability. We suggest relative cue contrast may provide an alternative explanation of these results.

### (a) Modelling landscape

Conceptually, none of the individual elements in the model we present are new [8,13,17,21,41]. Nevertheless, we are the first to provide a relatively complete implementation, and in particular, to combine adaptive synaptic connections for R-neurons with multimodal cue integration. Most previous models do not investigate multi-cue behaviour [8,20,28,42]. The closest work is provided by [43] (extended in [44]) which includes two populations of neurons with Gaussian activation profiles which input to an integration layer which itself forms a ring-attractor. Plasticity between the input and integration (compass) neurons is not included. This is noted as a strength of the model (conceptual simplicity), however, it does not reflect known phenomena in fruit flies and dung beetles [16,20,22]. Such rigidity also creates functional problems with spatially separated cues which automatically form conflicts unless their relationship is innately encoded.

### (b) Hebbian learning facilitates vector summation

Hebbian learning is often suggested for the construction of R → E-PG mappings [16,17,19,20,28,41]). We show here that such plasticity does not interfere with the vector summation properties of the circuit provided the learning is structured by being linked to periods of increased angular velocity [21,28]. We also note that, in order to build the relationship between perceived cue motion and angular velocity, cue input cannot drive the compass during learning. We found this to be true during model construction but it is also fairly intuitive; if R input drives the E-PG signal and R → E-PG connections are updated in a Hebbian fashion, the active R neurons will continually remap to the active E-PGs (see the electronic supplementary material, continuous learning). Other models have assumed that R neurons do not provide any input to the E-PGs during learning [28,42]. We instead suggest that the balance of influence between the self-motion and cue input in the E-PGs could be flexibly regulated by angular velocity in the same manner as the plasticity between R and E-PG neurons.

### (c) Cue contrast as a proxy for reliability

Previous work in insect cue integration has suggested that cue influence is determined according to reliability [7–9,22,26]. Our conflict simulations (figure 6) indicate that cue reliability, while affecting the formation of R-to-E-PG connections, does not have a significant effect on cue influence. Further, our behavioural results and accompanying simulations clearly indicate that cue weight is not determined by reliability for the dung beetle *K. lamarcki*, contrary to previous claims [9,22,26].

In previous dung beetle cue integration studies, it is not always clear that cue reliability was manipulated. Reliability estimation usually requires performing a large number of trials with the same individual, which is often impractical in insects. Khaldy *et al*. [26] manipulated the intensity of a sun cue but did not provide evidence that this significantly affected reliability of orientation. In [22] the assumption that higher sun elevations are less reliable is justified by reference to [24] in which the difference between two exit angles is taken to be a measure of orientation precision. In [9] the orientation precision (estimated from multiple exits) of a beetle under an ersatz sun did not appear to be reduced until very high elevations (> 80°). In this paper, sun elevations for which precision did not significantly differ nevertheless had different effects on cue integration: beetles orient to the sun for a 45° elevation but the wind for 75° elevation, which suggests reliability is not determining cue weight.

On the other hand, changing elevation or intensity, as done in these studies, would be expected to change the *contrast* of the sun cue. Indeed it is possible that the directional cue provided from the sun actually comes from intensity gradient it produces across the sky, which beetles are known to use for orientation [45–47]. Cue contrast can contribute to cue reliability by reducing detection error (figure 1), but (unlike reliability) is directly available in the instant. A neural circuit that has evolved to weight cues by contrast would in most natural situations obtain a good proxy of weighting by reliability.

### (d) Future work

#### (i) R neuron representation

A recent connectonomic analysis observed that R neurons inhibit all other R neurons in the same group (e.g. all R1 neurons inhibit all other R1 neurons) [13]. These connections were not included as the data was not available when the initial model was constructed. We plan to investigate whether within-group inhibition supports a sinusoidal activity profile which we assumed for R neuron encoding of cue direction (consistent with [19] and [8]) but which has not been directly shown. By analogy to the EB [13] inhibition could encourage a sinusoidal shape for different input profiles. A second possibility is that inhibition could provide a mechanism for reliability-based weighting, keeping the peak of an activity bump relatively stable, while modulating the amplitude as a result of noise (see [8]). R neuron groups also inhibit each other, which could implement a hard-wired, yet flexible cue hierarchy (which sufficient weighting could overcome); alternatively, this could partially implement our R activity normalization scheme.

#### (ii) Questions in dung beetles

As solar elevation increases, dung beetle orientation precision remains relatively stable then decreases rapidly at high elevations [9]. This is what we might expect if the contrast at lower elevations remains well above a detection threshold. A contrast assay [47] could be used to check what solar to anti-solar contrast corresponds to the minimum usable contrast threshold in *K. lamarcki* . It would then be useful to try and determine how the beetles compute solar to anti-solar contrast. There may also be a way to measure wind cue contrast by examining antennal displacement for different wind speeds (and directions).

While our data suggest that dung beetles do not fully weight cues by reliability, our modelling suggests that reliability could have some effect on cue influence. It would be useful to examine this explicitly in the animal, e.g. by adding azimuthal variation to a sun cue.

## 5. Conclusion

Cue integration research (our own included [9]) tends to assume Bayesian reasoning: a cue estimate includes information about the variance of the estimate (reliability), which is used to weight the cue [1,3]. However, the meaningfulness of a Bayes-optimal result is questionable [48] and the use of Bayesian modelling is disputed [49–53]. In this small, behavioural/modelling example, the Bayesian assumption was counterproductive; only by leaving reliability behind were we able to make sense of our data. Future behavioural data may be better understood by first asking what quality of a cue is actually changed by an experimental manipulation, rather than assuming reliability is involved. This could expose a host of efficient, elegant solutions to otherwise computationally intensive problems.

## Data Availability

All raw data and code are available in the public GitHub repository accompanying this paper (https://github.com/refmitchell/CX_cue_integration_model/tree/v1.0.0 [https://doi.org/10.5281/zenodo.7788258] [54]). Follow the link to access this repository and download our code/data. Additional experiments in our electronic supplementary material [55] use data previously published in Dacke *et al*. [22] and Shaverdian *et al*. [9] (used with author permission). This data is included in our repository for completeness (to allow others to replicate our analysis) and the sources are clearly marked. Software implementation details and additional modelling results can be found in the electronic supplementary material [55].
