# Correction to: ‘A model of cue integration as vector summation in the insect brain’ (2023) Mitchell *et al.*

**DOI:** 10.1098/rspb.2023.1993

**Published:** 2023-09-20

**Authors:** Robert Mitchell, Shahrzad Shaverdian, Marie Dacke, Barbara Webb

**Keywords:** vector, cue integration, plasticity, contrast, reliability, neural model

*Proc. R. Soc. B* **290**, 20230767 (Published online 28 June 2023). (doi:10.1098/rspb.2023.0767)

In the original version of this article [1], fig. 6 contained data which were based on a flawed simulation script. This script has been fixed in our code repository, and the corrected version of fig. 6 is included here. The data still support the original point that cue weight primarily determines cue influence. The authors apologize for any confusion which arose as a result of this minor error.

**Figure:**
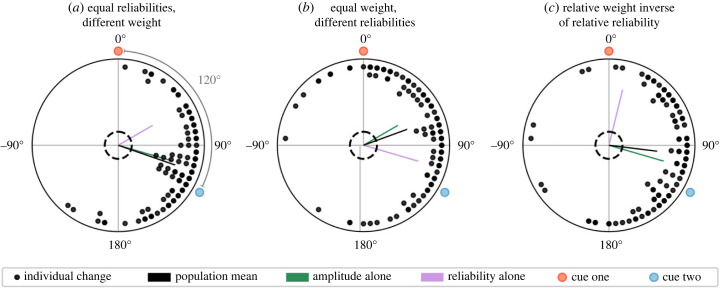
Replacement for fig. 6 in [1]. Effect of weight and reliability on cue influence in behavioural simulation of cue conflict. The population mean vector (black) falls closer to the theoretical vector sum where magnitudes are determined by cue weight (green) than where magnitudes are determined by reliability (magenta). Dashed rings indicate the threshold for significance using a Rayleigh test (*p* < 0.05).
